# Development of a 3D-Printed Dosing Platform to Aid in Zolpidem Withdrawal Therapy

**DOI:** 10.3390/pharmaceutics13101684

**Published:** 2021-10-14

**Authors:** Silke Henry, Lien De Vadder, Milan Decorte, Susanna Francia, Magali Van Steenkiste, Jan Saevels, Valérie Vanhoorne, Chris Vervaet

**Affiliations:** 1 Laboratory of Pharmaceutical Technology, Ghent University, 9000 Ghent, Belgium; Silke.Henry@UGent.be (S.H.); Lien.DeVadder@UGent.be (L.D.V.); Milan.Decorte@UGent.be (M.D.); Valerie.Vanhoorne@UGent.be (V.V.); 2 Laboratory of Pharmaceutical Technology, Università Di Pavia, 27100 Pavia, Italy; susanna.francia01@universitadipavia.it; 3 Algemene Pharmaceutische Bond, 1000 Brussels, Belgium; magali.vansteenkiste@apb.be (M.V.S.); jan.saevels@apb.be (J.S.)

**Keywords:** fused deposition modeling, 3D printing, personalised medicine, zolpidem, extrusion

## Abstract

The long-term use of benzodiazepine receptor agonists (BZRAs) is associated with multiple side effects, such as increased sedation, hangover or an elevated risk of dependency and abuse. Unfortunately, the long-term use of BZRAs is reaching worrying intake rates, and therefore, the need for action is high. It was demonstrated already that the overall willingness of patients for deprescription increased when a slow dose reduction scheme with the possibility for dose increase, if needed, is employed. The current study aims to develop a flexible dosing platform of zolpidem hemitartrate (ZHT) to facilitate such withdrawal therapy. As this is the first report on the extrusion and 3D printing of ZHT, its thermal behaviour and sensitivity towards photolytic degradation was characterised. It was shown that ZHT possesses multiple polymorphs and was especially prone to oxidative photolysis. Next, a variety of immediate release polymers (Eudragit EPO, Kollidon VA64, Kollidon 12PF and Soluplus) were blended and extruded with Polyox WSR N10 to investigate their feedability and printability by mechanical and rheological analysis. The addition of PEO was shown to enable printing of these brittle pharmaceutical polymers, although the processing temperature was deemed critical to avoid surface defects on the resulting filaments. An EPO(70)PEO(30) system was selected based on its suitable mechanical properties and low hygroscopicity favoring ZHT stability. The matrix was blended with 1% or 10% API. The effect of certain printing parameters (caplet size, nozzle diameter, % overlap) on dissolution behaviour and caplet weight/dimensions/quality was assessed. A flexible dosing platform capable of delivering <1 mg and up to 10 mg of ZHT was created. Either caplet modification (incorporation of channels) or disintegrant addition (Primojel, Explotab, Ac-Di-Sol, Primellose and Polyplasdone-XL) failed to achieve an immediate release profile. This study provides the first report of a 3D-printed flexible dosing platform containing ZHT to aid in withdrawal therapy.

## 1. Introduction

Benzodiazepine receptor agonist (BZRA) utilisation has reached worrying intake rates of 28 to 55% in European retirement facilities [1,2]. The PHEBE project investigated psychotropic intake in 76 Belgian nursing homes and found an alarming intake rate of 79%, of which 54% was attributed to BZRA use by residents. Insomnia was the main indication for chronic BZRA use, and lormetazepam (38%) and zolpidem (23%) were predominantly prescribed. The geriatric upper limit was exceeded in 82% of these cases for zolpidem [1]. Another study discovered that residents with worsening dementia likewise demonstrated an increased use of antipsychotics [3], although it is anticipated that these drugs might increase cognitive impairment [1]. In general, it was shown that 35.8% of patients on BZRA medication continue its use beyond 3 months, and that BZRA-related deaths have increased by a factor of more than 4 in recent years [4].

At the moment, 35 BZRAs exist which bind to the γ-aminobutyric acid type A receptor and increase its affinity for the inhibitory neurotransmitter GABA. The majority of BZRAs are longer-acting due to their pharmacologically active metabolites and are used for their anxiolytic or anticonvulsant effects. Short-acting BZRAs are mostly employed as hypnotic agents [4]. Short-term use of these BZRAs has shown to effectively lessen insomnia by reducing sleep onset time by approximately 4 min and increasing the overall sleep duration by 60 min [5]. Unfortunately, these beneficial effects are only short-lived (one day to six weeks) [6], and a reduction of treatment efficacy has been suggested after four weeks of consecutive BZRA use [7]. This diminished efficacy has been linked to a physical change of the γ-aminobutyric acid type A receptor; however, as efficacy decreases, the negative side effects might prevail [6]. These side-effects include an elevated risk of dependency, withdrawal symptoms and potential cognitive impairment. In the elderly, BRZAs might cause increased sedation, hangover and risk of falling [1]. Strikingly, BZRAs have also shown to release dopamine in the mesolimbic area, which is correlated to the action mechanism of drugs with risk of abuse. As a result, BZRA abuse is also associated with mental illness and drug overdosing [4].

Tighter regulation of BZRA prescriptions was suggested as a risk mitigation strategy, especially in the elderly population due to the evidence of potential harm. Treatment of existing BZRA abuse is a difficult task, as currently no medication is approved to aid in withdrawal. One of the strategies to treat these patients is deprescribing through education and dose reduction, which is a cost-effective process requiring limited resources [4,6,8]. In general, it is advised to discontinue the BZRA gradually over a period of 4 to 6 weeks to avoid serious withdrawal symptoms. It has been shown that sudden withdrawal mostly induces seizures, delirium and psychosis. Other frequent symptoms include, for example, symptom rebound, anxiety, muscle tension, panic disorders or perception disorders. Dose reduction schedules might range from a reduction of 50% each week to 10% every 2 weeks. Furthermore, switching to a long-acting BZRA (e.g., diazepam) has been suggested, since withdrawal from short-acting BZRAs was associated with higher drop-out rates. Unfortunately, this has not yet been associated with better outcomes. Blind reduction, where the patient is not aware of the actual dose, has also been suggested [4]. It should be noted that the term ‘deprescribing’ covers the whole process of a patient ceasing a drug, as it is often complicated and includes dose individualisation to improve patient adherence. Patient engagement and professional support by development and communication of a cessation regimen are vital. It was shown that a slow dose reduction with the possibility for a dose increase if withdrawal symptoms occurred improved the overall willingness of patients for deprescription [9]. In conclusion, BZRA abuse is a serious and frequently occurring condition which requires an individual, patient-tailored dosing regime to achieve deprescribing.

The on-demand production of a dosage form containing a personalised, flexible dose would greatly benefit this treatment strategy. Industrial manufacturing techniques (e.g., powder compaction) are cost-effective for mass production but are unable to produce such personalised dosage forms. Extrusion-based 3D printing, or fused deposition modeling (FDM), however, is a flexible, cost- and time-efficient technique on a small scale [10]. This technique utilizes a filament fed by roller grips to a heated nozzle, where the filament softens and is subsequently deposited via printing into a specific shape. The feedstock material should possess unique mechanical and rheological characteristics to enable successful 3D printing. The exact processing conditions (e.g., print speed, nozzle size, temperature) often depend on a combination of these filament properties [11]. One of the key properties to ensure feedability is the mechanical behaviour of the filament, as it should possess mechanical resilience in order to avoid breaking or deforming on the feeding gears [12,13]. Unfortunately, hydrophilic pharmaceutical polymers are often poorly printable due to their brittle nature. Improving their feedability can be achieved using either plasticizers, inert fillers or polymers with high strengths or by adaptation of the feeding mechanism [14,15]. The feedstock material used in FDM 3D printing consists mainly of a thermoplastic polymer with dispersed API, mostly produced using hot melt extrusion (HME) [16]. When the feedstock is prepared and proves to be feedable and printable, a 3D object is created by moving the nozzle or bed into different axes [17]. This combination of HME with FDM 3D printing has been proven successful to produce a variety of drug delivery systems, like oral dosage forms, implants, intrauterine systems or veterinary applications [18]. In terms of oral dosage forms, both conventional tablets or caplets have been prepared [19,20,21], as well as exotically shaped/sized tablets which exploit the flexibility of the 3D printing technique [22,23,24,25].

In this study, zolpidem hemitartrate will be used as a BZRA to create a 3D-printed flexible dosage form to aid in withdrawal therapy. The stability of this API was already investigated by forced degradation studies [26,27], although only a limited number of studies utilizing ZHT to produce dosage forms was reported. Moreover, previous studies focused mainly on either increasing the drug dissolution rate or developing extended-release tablets, which were thought to be beneficial in terms of efficacy and patient compliance by supporting sleep maintenance [28,29]. To our knowledge, ZHT was not investigated previously to develop a flexible dosing platform to aid in withdrawal therapy. The present paper is also the first report of utilizing ZHT in a FDM 3D printing process.

## 2. Materials and Methods

### 2.1. Materials

Zolpidem hemitartrate hemihydrate (Aarti Drugs, Mombasa, India) was used as the active pharmaceutical ingredient (API) to produce a 3D-printed dosing platform. The solubility of ZHT is pH dependent and lies around 7–8 mg/mL at a pH of 5.8 [30]. Hydrophilic polymers (Eudragit EPO, Kollidon VA64, Kollidon 12PF, Soluplus and Polyox WSR N10) were investigated as immediate release matrices. Eudragit EPO was kindly gifted by Evonik (Hanau, Germany) and is an amorphous polymer with a glass-transition temperature of 52 °C. It is a methacrylate copolymer with an approximate molecular weight of 47,000 g/mol. Kollidon VA64, Kollidon 12PF and Soluplus were kindly gifted by BASF (Ludwigshafen, Germany) and are amorphous polymers with glass-transition temperatures of 101 °C, 90 °C and 70 °C, respectively. Kolldion VA64 is a vinylpyrrolidone/vinyl acetate copolymer in a 60/40 ratio with an approximate molecular weight of 45,000 g/mol. Kollidon 12PF is a polyvinylpyrrolidone polymer with an approximate molecular weight of 2500 g/mol. Soluplus is a polyvinylcaprolactam-polyvinyl acetate-polyethylene glycol graft copolymer in a 13/57/30 ratio with an approximate molecular weight of 118,000 g/mol. Polyox WSR N10 was kindly gifted by Dupont (Hamm, Germany) and is a semi-crystalline polymer with a glass-transition and melting temperature of −67 °C and 65 °C, respectively. It is a non-ionic polyethylene oxide with an approximate molecular weight of 100,000 g/mol. Scotch blue painter’s tape, 50 mm, was supplied by 3M (Bracknell, UK).

The following disintegrants were added to certain polymeric blends: sodium starch glycolate (Primojel or Explotab), croscarmellose sodium (Ac-Di-Sol or Primellose) and crosslinked polyvinylpyrrolidone (Polyplasdone-XL). Ac-Di-Sol was supplied by Dupont (Wilmington, Delaware, USA), Primellose and Primojel by DFE pharma (Goch, Germany), Polyplasdone-XL by Ashland (Schaffhausen, Switserland) and Explotab by JRS (Rosenberg, Germany).

### 2.2. Zolpidem Hemitartrate: Physico-Chemical Characterization

#### 2.2.1. Thermogravimetric Analysis

Thermogravimetric analysis (TGA 2950, TA instruments, Leatherhead, UK) was performed on ZHT under a dry nitrogen flow (100 mL/min). The sample (±7 mg) was first equilibrated at 30 °C and was subsequently heated to 300 °C using a heating rate of 10 °C/min.

#### 2.2.2. Differential Scanning Calorimetry

Differential scanning calorimetry (DSC) was performed on ZHT to determine the thermal properties of the API. The analysis was performed using Tzero pans (TA instruments, Belgium) in a DSC Q2000 (TA Instruments, Leatherhead, UK) using a dry nitrogen flow rate of 50 mL/min. A heat-cool-heat run at heating/cooling rate of 10 °C/min was applied from 20 to 200 °C.

#### 2.2.3. Hot Stage Microscopy

The melting and recrystallization of ZHT was visualised and compared to lidocaine hydrochloride monohydrate (Fagron, Nazareth, Belgium) using a hot stage microscope (BX51, Olympus, Hamburg, Germany). Lidocaine undergoes melting without crystallization during heating; hence, the different behaviour of ZHT can be illustrated. A heat ramp from 20 to 120 °C at a rate of 10 °C/min was imposed using the Linksys 32 software (Linksys 32, Linkam, Surrey, UK). The samples were visualised and their behaviour recorded using a digital camera mounted on top of the microscope.

#### 2.2.4. Raman Spectroscopy

Raman spectroscopy was performed on zolpidem before and after heat treatment (160 °C, 5 min). Raman analysis was performed using a Raman Rxn 1 Microprobe (Kaiser Optical Systems, Ann Arbor, MI, USA) equipped with a 10× objective lens and air-cooled CCD detector. The laser wavelength of the NIR diode laser was 785 nm. Raman spectra were recorded in the range from 250 to 1890 cm^−1^ at 1 cm^−1^ resolution using a laser power of 400 mW and an exposure time of 10 s. Data analysis was performed using SIMCA (version 17.0, Umetrics, Umea, Sweden). Pre-processing using SNV and AsLS baseline correction was applied on the spectra before they were directly compared.

#### 2.2.5. X-ray Diffraction

X-ray diffraction (XRD) was performed on zolpidem before and after heat treatment (160 °C, 5 min). XRD analysis was performed using a D5000 Cu Kα diffractor (λ = 0.154 nm) (Siemens, Karlsruhe, Germany) with a voltage of 40 mV in the angular range of 10° < 2θ < 60° using a step scan mode (step width = 0.02°, counting time = 1 s/step).

### 2.3. Zolpidem Hemitartrate: Oxidative Potential

Forced degradation by photolytic oxidation was performed on ZHT using a method as described by Malesevic et al [27,31]. Degradation of the API in solution and solid state in the presence or absence of light was investigated using HPLC-UV analysis. A LiChrospher 100 RP-18 (5 μm) 250 × 4.6 mm (Merck, Darmstadt, Germany) column was used. The mobile phase consisted of methanol:10 mM ammonium acetate (68:32, v/v) with pH adjusted to 5.4 using glacial acetic acid. A HPLC system (Merck, Darmstadt, Germany) coupled with a UV detector (type L-7400) was used. The method has a limit of detection of 0.07 μg/mL and limit of quantification of 0.24 μg/mL for ZHT. 50 mg of ZHT was dissolved in 25 mL of a methanol:water mixture (30:70). 2 mL of this stock solution was diluted with 2 mL water and sample solutions were stored in hermetically sealed glass vials for either 3 or 7 days in the presence of light. Dark controls were prepared accordingly but stored for either 3 or 7 days in the refrigerator, protected from light. 0.4 mL of these sample solutions were diluted with 0.6 mL of the mobile phase and injected in the HPLC system. Alternatively, photo-induced oxidation in the solid state was also investigated to determine whether specific packaging is needed for the drug product. Dry powder samples were subjected to light during 7 days (40% RH atmosphere) or 3 months (hermetically sealed glass vial). Prior to HPLC analysis, a 50 mg sample was weighed and diluted as described previously.

### 2.4. Filament Preparation: Hot Melt Extrusion

#### 2.4.1. Placebo Filaments

Matrix systems consisting of Eudragit EPO, Kollidon 12 PF, Kollidon VA64 and Soluplus were blended with Polyox WSR N10 in a 70:30 or 30:70 ratio and extruded using a co-rotating, fully intermeshing twin-screw extruder (Prism Eurolab 16, Thermo Fisher, Karlsruhe, Germany) equipped with co-rotating twin screws and a custom-made heated die of 1.70 mm diameter. A DD flex-wall 18 feeder (Brabender, Duisburg, Germany) was used. Screw speed and feed rate were kept constant at 40 rpm and 0.3 kg/h, respectively. A standard screw configuration consisting of transporting elements, two kneading blocks and a discharge element were used [32].

The processing range for HME of a specific polymer (blend) depends on its complex viscosity $\eta^{*}$, which should fall between 1000 and 10,000 Pa.s. Within this range, the torque limit of the extruder is not exceeded, while its mixing capability is guaranteed [33]. Different extrusion temperatures ranging from 120 to 180 °C were screened to find the ideal extrusion temperature. After HME, the filaments were collected on a self-winding roller and the roller speed was adapted to obtain filaments with a diameter of 1.75 ± 0.05 mm as measured with a digital caliper. The roller was necessary due to die swelling, which is an increase of the filament diameter upon leaving the die. Die swell occurs due to relaxation of the polymers after exposure to high stress within the extruder and die, and the extent depends on HME parameters and intrinsic polymer properties [34]. Filaments with a diameter outside the acceptable range for printing were discarded.

#### 2.4.2. Drug-Loaded Filaments

A matrix system of EPO:PEO (70:30) was chosen as the preferred carrier due to the unique properties of ZHT and the requirements to achieve a 3D-printable formulation. Drug-loaded filaments containing 1 or 10% of API and 4, 8, 12 or 16% of disintegrant were extruded using the same method as the placebo blends with another feeder (Coperion QT-20, Niel, Belgium). Extrusion and die temperature were 140 and 120 °C, respectively.

### 2.5. Filament Characterization

#### 2.5.1. Raman Microscopy

The surface of Kollidon VA64:Polyox WSR N10 (70:30, 30:70) was investigated using light and Raman microscopy due to the occurrence of surface defects at certain process temperatures. The same Raman method and pre-processing techniques were used as described for the ZHT analysis but with increased exposure time (15 s). Principal components analysis (PCA) using SIMCA (version 17.0, Umetrics, Umea, Sweden) was performed on pre-processed spectra, which is a technique used to characterize a data set by maximizing its variation [35].

#### 2.5.2. Mechanical Properties

Mechanical properties of the extruded placebo and drug-loaded filaments were investigated within 24 hours after HME. Samples were subjected to a tensile test in elongation mode using a TA.HD PlusC Texture analyser (Stable Micro Systems, Surrey, UK) equipped with pneumatic clamps as described previously [11]. The specimen of 25 mm was elongated at a rate of 0.02 mm/s until 20% strain was reached. Using Matlab2018b, the Young’s modulus, strain and stress at break and tensile energy to break the filament (area under the curve) were calculated as an average of fifteen independent samples. The Young’s modulus was calculated as the slope between 0.05 and 0.25% strain in the stress-strain curve [36].

#### 2.5.3. Rheological Analysis

Rheological analysis by means of a temperature sweep was performed on the pure polymers and extruded placebo/drug-loaded filaments to determine the temperature dependency of viscosity. A stress-controlled HAAKE Mars III rheometer (Thermo Scientific, Karlsruhe, Germany) equipped with a parallel plate geometry of 20 mm diameter and a Peltier temperature module was used. After zero gap determination at the test temperature, samples were loaded and allowed to soften. The sample was trimmed and excess material was removed at a gap size of 1.1 mm. Samples were equilibrated at the measuring gap (1 mm) prior to testing. Temperature sweeps were performed monitoring $\eta^{*}$, $G^{\prime }$ and $G^{\prime \prime }$ in function of temperature under a constant frequency of 6.28 rad/s. Samples were molten and equilibrated at 180 °C, followed by a cooling run at 2 °C/min to 80 °C at a strain deformation of 1%, which proved to be within the linear viscoelastic region [11].

Frequency sweeps were performed on drug-loaded filaments at 160, 150, 140, 130 and 120 °C using a strain deformation of 1%. It should be noted that the deformation of 1% is within the linear viscoelastic regime for the frequency of 6.28 rad/s, but the non-linear regime is also assessed, as the frequency is increased to larger values during the frequency sweep. The storage modulus ($G^{\prime }$), loss modulus ($G^{\prime \prime }$) and dynamic viscosity ($\eta^{*}$) of the frequency sweeps were shifted to the frequency sweep of 160 °C using the TTS module of the HAAKE Rheowin software, resulting in a temperature-invariant mastercurve. From the obtained shift factors (aT), the Arrhenius flow activation energy (kJ × K${}^{-1}$ × mol${}^{-1}$) was calculated, as shown in Equation (1) [37]:

(1) $$E_{a}=\frac{R_{G}\ln aT}{\frac{1}{T}-\frac{1}{T_{R}}}$$

where *R* is the gas constant of 0.008314 kJ × K${}^{-1}$ × mol${}^{-1}$, aT is the horizontal shift factor for a frequency sweep recorded at temperature *T* and *T*${}_{R}$ is the reference temperature at which the mastercurve is created.

The Cross model, as shown in Equation (2) [38], was fitted to the temperature-invariant mastercurve to to determine the impact of the API on the rheological properties of the filament.

(2) $$\eta^{*}\left(\dot{\gamma },T\right)=\frac{\eta_{0}}{1+{\left(\frac{\eta_{0}\dot{\gamma }}{\tau^{*}}\right)}^{\left(1-n\right)}}$$

where $\tau^{*}$ is the critical shear stress at which the complex viscosity profile moves from Newtonian to shear thinning, *n* is the power-law index which accounts for the degree of shear-thinning and $\eta_{0}$ is the zero-shear viscosity.

#### 2.5.4. Karl Fischer Titration

The moisture content (*n* = 3) of placebo filaments was investigated by Karl Fischer titration (Mettler Toledo, Schwerzenbach, Switzerland). Within 24 hours after HME, crushed filament was dissolved in a dry methanol:formamide (2:1) mixture and titrated with Hydranal-Composite 5 titrant solution. Pieces of filaments were stored for one week at RH 60% and 25 °C, after which a new moisture analysis was performed to determine how much moisture was absorbed after exposure to a humid environment.

#### 2.5.5. Content Uniformity

Content uniformity analysis was performed on drug-loaded filaments to ensure the API was uniformly dispersed within the filament, even at low drug loads (1%). Independent samples of 100 mg (*n* = 10) were weighed, crushed and dissolved in 0.01 M HCl. The solution was filtered and diluted, its absorbance was measured at 295 nm using a UV-1650PC spectrophotometer (Shimadzu, Brussel, Belgium) and the API content compared to the theoretical content was determined using a calibration curve.

### 2.6. Caplet 3D Printing

Extruded filaments were stored in a dessicator and protected from light in between 3D printing experiments. Caplets with 11 mm length, 5.5 mm height and 4.4 mm width were designed as a .stl file using OnShape and converted into G-codes using Slic3r Prusa Edition software (Prusa Research, Prague, Czech Republic). The following settings were employed: 0.1 mm layer height, 20% line infill, 1 shell, 1 top and 2 bottom layers. The extrusion multiplier was set to 1. The first layer of the caplet was printed with a speed of 10 mm/s, and other layers were printed at a speed of 50 mm/s. A fan, blowing on the printed object, was disabled during the first layer and enabled at 100% of its maximum speed during the consecutive layers. All caplets were printed with a nozzle temperature of 160 °C and bed temperature of 55 °C. Most caplets were printed with a nozzle size of Ø0.25 mm and an overlap of 50%; however, the influence of nozzle size and % overlap on certain caplet properties was also investigated by comparing different nozzle sizes (Ø0.25 mm, 0.4 and 0.6 mm) and different % overlaps (10, 30, 50%). Divergent caplet shapes were also created: an enlarged caplet with a size of 19.2 × 9.6 × 7.68 mm (90% infill) and a caplet with 2 rows and 5 columns of 0.6 mm square-shaped channels.

### 2.7. Caplet Characterization

#### 2.7.1. Mass, Dimension and Hardness Analysis

Mass and dimensions of the 3D-printed caplets (*n* = 10) were recorded using an automated tablet tester (SmartTest 50, Sotax, Basel, Switzerland). The breaking force was also determined for caplets (*n* = 10) prepared for nozzle comparison.

#### 2.7.2. Helium Pycnometry

The porosity of caplets (*n* = 3) prepared for nozzle comparison was calculated based on helium pycnometry measurements.

(3) $$Porosity\left(\%\right)=\left(1-\frac{V_{app}}{V_{theo}}\right)*100$$

where $V_{app}$ represents the apparent volume as determined by the helium pycnometer (AccuPyc 1330, Micrometrics, Norcross, GA, USA) at an equilibration rate of 0.00050 psig/min with number of purges set to 10. $V_{theo}$ represents the theoretical volume of the caplet (212.17 mm${}^{3}$), as determined by the PrusaSlicer software.

#### 2.7.3. In-Vitro Dissolution

In vitro release experiments were based on the USP guidelines for ZHT immediate release tablets (Dissolution <711>, Test 1). The acceptance criterium is that not less than 80% of the labeled amount of zolpidem tartrate is dissolved within 15 min. A PTWS 120D dissolution system (Pharma test, Hainburg, Germany) with a rotational speed of 50 rpm was used. The dissolution medium was 900 mL of sonicated 0.01M HCl solution, of which 5 mL samples were withdrawn at each time point and analysed spectrophotometrically (Shimadzu, Brussel, Belgium) at 295 nm. Three different dissolution protocols were tested, because the printed caplets floated in the medium: a paddle apparatus with or without sinker and a basket apparatus. Use of a sinker proved to be the most repetitive method and was used for all remaining dissolution tests. For each dissolution run, one vessel contained a ZHT 10 mg (EG, Brussel, Belgium) tablet to monitor the quality and consistency of the proposed dissolution method.

## 3. Results and Discussion

### 3.1. Zolpidem Hemitartrate

ZHT possesses multiple polymorphs with different X-ray powder diffraction (XRD) patterns, water contents and thermal properties [39]. The polymorph used in the current study and its conversion upon heating will be described, along with its photolytic degradation properties.

#### 3.1.1. Physico-Chemical Characterization

**Figure 1:**
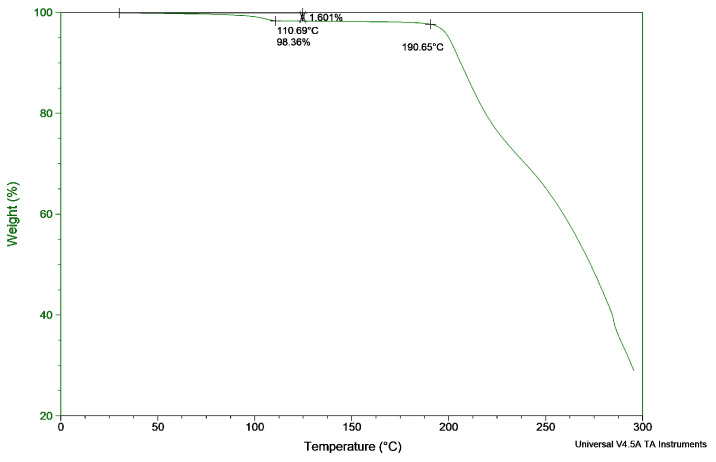
Thermogravimetric analysis (TGA) of zolpidem hemitartrate (ZHT) showing a weight loss of 1.6% at 110.69 °C (loss of water) and a gradual weight decrease starting at 190.65 °C.

**Figure 2:**
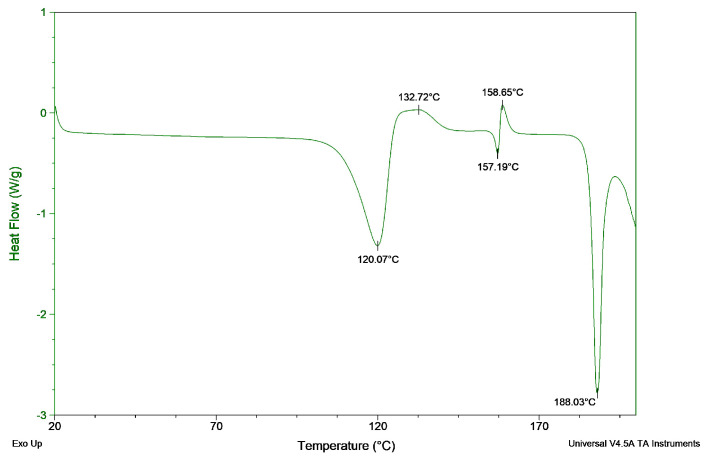
Differential scanning calorimetry (DSC) analysis of zolpidem tartrate showing an endothermic peak at 120.07 °C, a broad exothermic peak at 132.72 °C, a fast polymorphic transition at 157.19 °C and 158.65 °C and finally a melting peak at 188.03 °C, after which degradation is initiated.

ZHT was subjected to a heating run by thermogravimetric analysis, as can be seen in Figure 1. A small weight loss of 1.6% occurred at 110.69 °C, which was associated with a loss of water. A gradual, continuous weight decrease started from 190.65 °C. DSC analysis (Figure 2) during a heat run revealed an endothermic peak at 120.07 °C, a broad exothermic peak at 132.72 °C and a fast polymorphic transition at 157.19 °C and 158.65 °C. Finally, an endothermic peak associated with melting occurred at 188.03 °C, after which degradation was initiated.

The first endothermic peak at 120.07 °C in DSC was correlated with the first step change in the TGA profile at 110.69 °C and corresponds to a loss of water (dehydration endotherm) bound within the crystal lattice. Energy is required to break the intermolecular forces between the solid drug and the crystallization solvent, yielding a desolvated solid. Often the desolvated solid subsequently rearranges its molecular bonds to create a more stable polymorph [40]. This is an exothermic process which releases energy and occurs at 136.16 °C for ZHT. This dehydration and restructuring process was visualised using hot stage microscopy (HSM) and compared with the melting process of lidocaine hydrochloride monohydrate, which is a hydrate crystal with a single melting peak around 82 °C. By comparing both APIs in the hot stage microscope, any melting of zolpidem occurring at ±120 °C could be excluded. This video material can be found in the Supplementary Materials.

**Figure 3:**
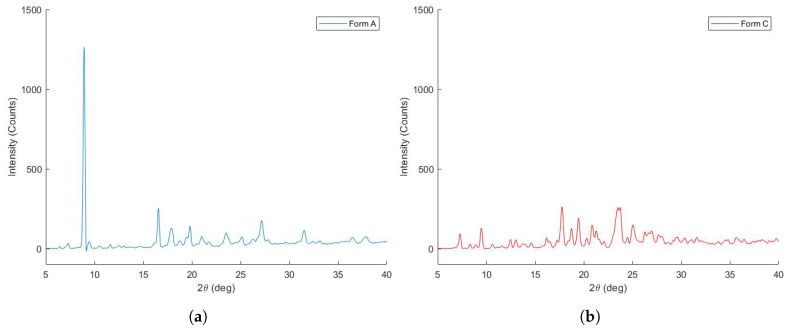
XRD profiles of zolpidem tartrate polymorphs before and after heat treatment, which were identified as zolpidem hemitartrate form A (hemihydrate) (**a**) and form C (**b**), respectively.

**Figure 4:**
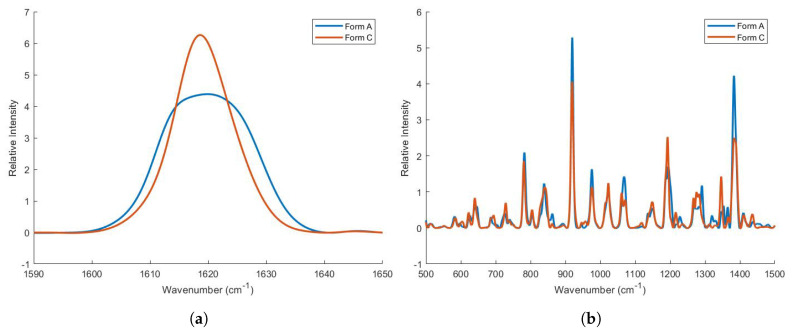
Differences in the Raman spectra of two polymorphs of zolpidem hemitartrate (form A and form C). (**a**) The intense band representing the C=O stretching of the amide showed band broadening in the hydrate crystal (form A) due to hydrogen bonding. (**b**) Both spectra are fairly divergent, which indicates a distinct re-arrangement within the molecule upon dehydration.

Due to the heating process, ZHT was converted from crystalline form A to form C, another stable polymorph. This was assessed during a DSC heat-cool-heat run, as there was no recrystallization peak present in the cooling run after heating to 160 °C. Moreover, in the second heating run, only the endotherm at 188.06 °C remained, and no polymorphic transitions/dehydration processes occurred. A comparison between both polymorphs was made using XRD and Raman spectra of zolpidem before and after heat treatment (5 min., 160 °C). Zolpidem before heat treatment possessed the characteristic XRD peaks at 6.40, 8.92, 11.60, 16.20, 16.56, 17.24, 24.46, and 27.16 ± 2θ of form A, as can be seen in Figure 3. Zolpidem after heat treatment possessed XRD peaks at 7.28, 9.48, 10.64, 12.48, 13.04, 13.72, 14.60, 16.20, 17.76, 18.76, 19.48, 20.32, 21.28, 23.52, 23.76, 25.04 and 26.96 ± 2θ. Zolpidem before and after heat treatment was identified as ZHT form A and form C, respectively, based on their XRD and thermal profiles [39]. These different polymorphs were also characterised using Raman spectroscopy and showed considerable differences in terms of peak width, intensity and peak positions. Raman spectra of dry powders might reveal vibrational differences between polymorphs. In particular, C=O, N-H and C-H groups are strongly affected upon hydration and these spectral bands display broadening, intensification and/or a red shift upon hydrogen bond formation [41,42]. The intense band at 1620 cm${}^{-1}$ (C=O stretching of the amide) [43] is displayed in Figure 4 and showed band broadening in the hydrate crystal (form A). Furthermore, bound crystal water within the lattice might hinder vibrational or rotational movement, causing some bands to become Raman inactive [42]. Raman spectra differed mainly in the 500–1500 cm${}^{-1}$ region (skeletal vibration, fingerprint area), as can be seen in Figure 4. In conclusion, both spectra are fairly divergent, which indicates a distinct re-arrangement within the molecule upon dehydration [44].

#### 3.1.2. Photolytic Degradation

In the literature, ZHT in solution was found to be unstable towards acidic/alkaline hydrolysis and photolysis but stable towards neutral hydrolysis. Photolysis yielded oxozolpidem, zolpyridine and zolpaldehyde as major degradants. The solid form proved relatively stable, although photolytic degradation also occurred to a minor extent. Film coating of tablets inhibited this degradation pathway [27]. Unfortunately, 3D-printed tablets are not film coated, and as such, it is necessary to confirm the importance and to quantify the extent of the photolytic degradation pathway. Therefore, both ZHT solutions and dry powder were subjected to light and analysed by means of HPLC-UV (Table 1). ZHT solutions quickly turned yellow upon storage when exposed to light, which was confirmed by the presence of multiple degradants in these samples. Major degradants were zolpacid (RRT 0.85) and zolpyridine (RRT 2.05), along with two unidentified degradants. These degradants most likely have oxygen bonded to the ZHT molecule on pyridine, forming N-oxide, or on the lateral methyl groups, forming methoxy groups, based on the DAD spectra and LC-MS data from Malsevic et al [27]. Their presence suggests a photolytic oxidation pathway causing zolpidem degradation in addition to hydrolysis. This type of oxidative degradation was not tested during the forced degradation studies executed by Malesevic et al., as they used a 10% H_2_O_2_ solution to determine the oxidative potential of the drug and, therefore, only investigated the extent of nucleophilic/electrophilic oxidation. Strikingly, ZHT is also prone to photolytic degradation in a solid state, especially when stored in contact with the ambient atmosphere. This was discovered when drug-loaded filaments showed discoloration after exposure to light. Degradation of the drug substance was investigated to determine if specific packaging was required to ensure drug product stability during storage. The recovery of a sample stored within a hermetically sealed glass vial for 3 months (96.92%) was higher than a sample stored in contact with a 40% relative humidity (RH) ambient atmosphere for 7 days (96.01%). This recovery is the amount of remaining zolpidem in the sample, which was calculated using a calibration curve based on the peak area. This enhanced degradation was also confirmed visually, as the powder stored at ambient atmosphere quickly turned yellow. As mentioned before, ZHT is prone to both acidic and alkaline hydrolysis, but stable towards neutral hydrolysis [27]. Hydrolysis due to a high relative humidity environment is therefore unlikely to be the main reason for the higher degree of degradation of the solid samples of ZHT. However, as the sample in contact with the ambient atmosphere for 7 days was stored in plastic bags, oxygen could still diffuse through the packaging, which was not the case for the samples stored for 3 months in hermetically sealed vials [45]. This could cause photolytic oxidation, where UV light activates an autoxidative chain reaction between the drug molecule and molecular oxygen. The slight yellow discolouration seen in the 3 month samples can be attributed to the oxygen already present in the vial before closure. A high RH can speed up the oxidation reaction, as the molecular mobility within the solid powder is potentially increased [46]. Photolytic oxidation is not a new phenomenon. Other APIs, such as tetracycline and losartan, show a sensitivity towards oxidation once exposed to light [47,48]. UV light can cleave C-N bonds and oxygen (or singlet oxygen, which is formed through photosensitization of ground state molecular oxygen) reacts with the formed drug radicals to continue the oxidation process [49].

The suggestion of photolytic oxidation is further strengthened when taking a closer look at the structure of ZHT. During the initiation phase of the autoxidation chain reaction, a hydrogen atom is removed from the drug molecule. The benzyl group of zolpidem is prone to this hydrogen abstraction due to the formation of a relatively stable benzylic radical. The enamine in the imidazopyridine ring is another one of the labile sites within the drug molecule. This structure is photosensitive and can easily react with singlet oxygen to further decompose the drug [27]. The mitigation strategy for photolytic oxidation is multi-factorial, with protection from light being the most important factor. This can be achieved via appropriate packaging of the 3D-printed caplet. However, once the autoxidation chain reaction has started with the residual oxygen inside the package, packaging alone is not sufficient to completely inhibit or stop the oxidation process. It will only inhibit initiation of future oxidation processes. In this case, antioxidants can be added to the formulation to scavenge free radicals or quench oxygen. This will allow oxidation reactions to be terminated before the drug undergoes decomposition [45].

### 3.2. Screening and Characterization of Placebo Filaments

All polymers (Kollidon VA64, Kollidon 12PF, Soluplus, Eudragit EPO) were blended in a 70:30 or 30:70 ratio with Polyox WSR N10 to enable 3D-printing of these brittle polymer matrices. Their potential as 3D-printable matrix systems was investigated based on their mechanical/rheological properties and hygroscopicity.

#### 3.2.1. Extrusion Defects

**Figure 5:**
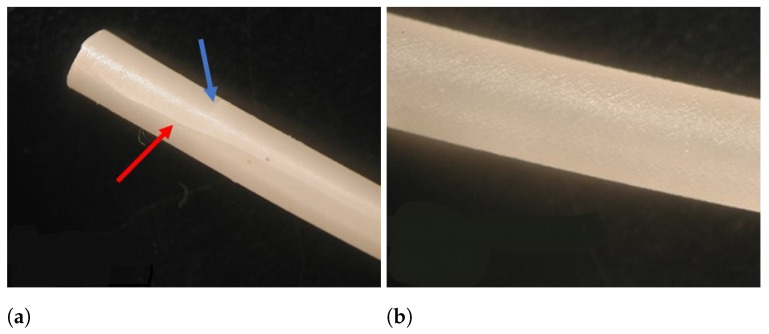
(**a**) Visualization of longitudinal and transversal fissures on the surface of a Kollidon VA64:PEO (70:30) blend processed at 140 °C and (**b**) absence of these extrusion defects for a Kollidon VA64:PEO blend (30:70) at 140 °C.

All placebo blends were extruded at 120, 140, 160 and 180 °C. Melt fracture with resulting fissures and defects was observed on the surface of the resulting extrudates for Kollidon VA64:PEO (70:30) processed at 120 and 140 °C, Kollidon VA64:PEO (30:70) processed at 120 °C and Kollidon 12PF:PEO (70:30, 30:70) processed at 120 °C. These fissures were present in the longitudinal and transversal direction (Figure 5) and are often denominated as ‘fir tree’ cracking defects due to melt fracture [50,51]. It is important to briefly investigate this phenomenon, since polymer blending is often suggested to enable 3D printing of brittle pharmaceutical materials [13,14,52]. Moreover, these extrusion defects hamper filament feedability, as will be discussed in the next section.

**Figure 6:**
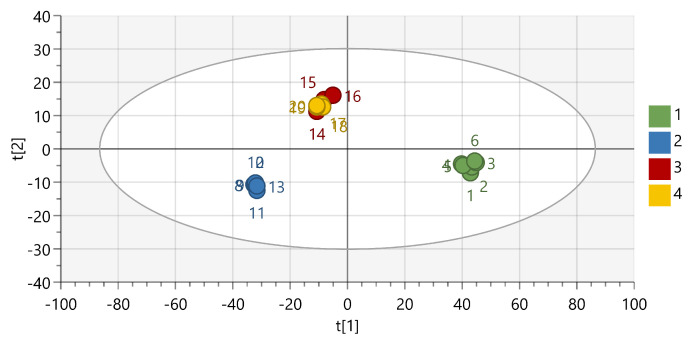
PCA of the full Raman spectra for PEO (class 1, green), Kollidon VA64 (class 2, blue), and KVA64:PEO (70:30, 140 °C) filaments near fissures (class 3, red) and smooth parts of the same KVA64:PEO filament (class 4, yellow).

**Figure 7:**
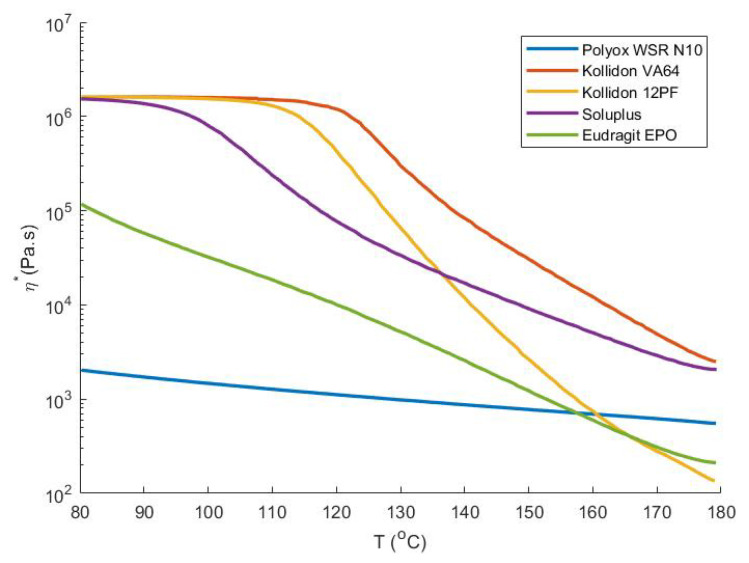
Complex viscosity as a function of temperature during a temperature sweep.

To investigate the origin of these defects, both Raman and rheological analysis were made. Raman spectra were recorded of different regions from the filament of Kollidon VA64:PEO (70:30) extruded at 140 °C. Principal component analysis was performed on the dataset including the full spectra, which resulted in a model containing 2 principal components (R2X: PC1 0.885, PC2 0.107) which explained 99.2% of the variation (Figure 6). The first principal component (PC) discriminates between the composition of the filament, as Kollidon VA64-containing samples are located on the negative PC1, which is different than the PEO samples. The second PC discriminates between the pure and blended filaments. Both clusters represent spectra recorded on either a fissure or a smooth part of the filament and seem to coincide with each other. Moreover, their location on PC1 reveals that both polymers (Kollidon VA64, PEO) are blended nicely on the investigated spots. As a result, the filament has a homogeneous composition over the whole filament surface and as such, defects do not arise from blending issues but are the result of excessive die wall shear stress, disturbing the melt flow. This was confirmed when analysing the viscosity over temperature profiles (Figure 7) of the pure polymers revealed a considerable viscosity difference of more than 10${}^{4}$ Pa.s between Kollidon VA64 and PEO at low temperatures (140 and 120 °C). It is also known that Soluplus seldom displays melt fracture, even under harsh extrusion conditions, which is supported by the presented data [53]. The presence of fissures for Kollidon 12PF:PEO at 120 °C and the absence of these defects for Eudragit EPO:PEO and Soluplus:PEO blends reveals the importance of reducing the die shear by using a higher die landing temperature, hence reducing the viscosity to obtain a high-quality end-product. Another remedy to avoid melt fracture could be to use a wider die or lowder extrusion rate, both of which are less ideal in the production of 3D-printable feedstock material. It is known that the critical stress at which melt fracture occurs is inversely correlated with the molecular weight (MW), with a limited effect of the molecular weight distribution. This MW dependency additionally explains why melt fracture was observed at a higher temperature for Kollidon VA64 when compared to Kollidon 12 PF. This is in contrast with shark skinning defects, as high-viscosity polymers with narrow molecular weight distributions are especially prone to this defect [51].

In conclusion, the reduced surface quality of the extrudate is assumed to originate from excessive friction within the die. High tensile stresses at the die exit have shown to induce cracking of the fluid, a phenomenon occurring especially for moderately or highly entangled polymers with higher viscosities [54]. The extrusion defect arising from viscosity mismatch was resolved by increasing the processing temperature, hence reducing the viscosity difference. These results reveal that care should be taken to optimize process settings when using polymer blends with large viscosity differences.

#### 3.2.2. Mechanical Properties

The feedability of the hydrophilic polymers (Kollidon VA64, Kolldion 12PF, Eudragit EPO, Soluplus) employed in this study was investigated previously [11] and these filaments were labeled as non-feedable due to their brittle nature. Mechanical properties of the filaments (stress and strain at break, tensile energy to break the filament) are predictive for such brittle feedability failure while the elastic modulus is more relevant for elastic filaments [11]. Addition of Polyox WSR N10 to these polymers, however, enabled their printing. Incorporating more PEO in the blend raised the stress and strain at breaking point, as well as the tensile energy required to break the filament (Table 2). For example, the addition of PEO to Eudragit EPO increased the stress required to break the filament from ±5 MPa (0% PEO [11]) to 14.0 MPa (30% PEO) and 20.9 MPa (70% PEO). Likewise, strain at break and the tensile energy to break the filament changed from ±2% and less than 10 × 10${}^{4}$ J/m${}^{3}$ (0% PEO [11]) to 4.3% and 36.0 × 10${}^{4}$ J/m${}^{3}$ (30% PEO) and finally to 4.9% and 57.0 × 10${}^{4}$ J/m${}^{3}$ (70% PEO). This beneficial effect of PEO addition on the mechanical properties and feedability of filaments is in accordance with literature [23].

**Figure 8:**
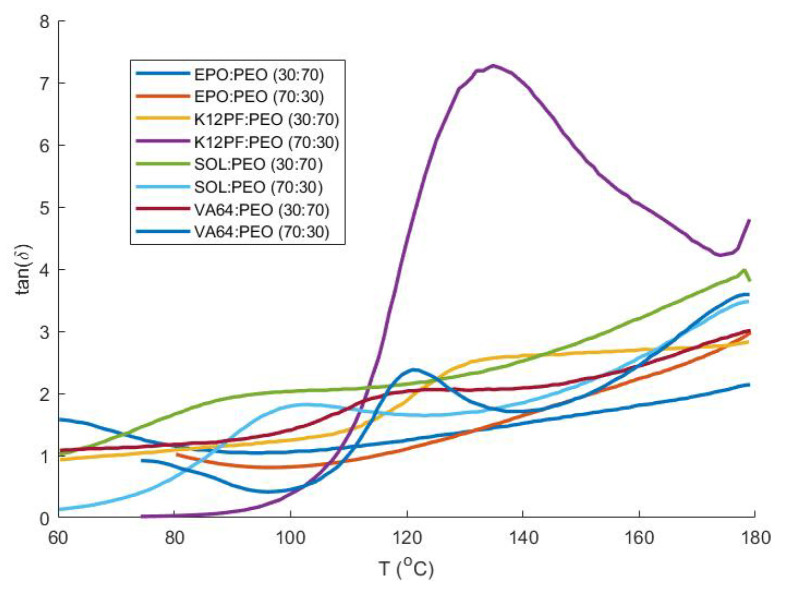
Dynamic temperature sweep of the loss tangent (tan δ) for all placebo blends, indicating a large peak in the Kollidon 12PF:PEO blend (70:30).

As discussed previously, certain blends were processed at different temperatures, yielding filaments with or without extrusion defects (fissures). Filaments displaying these fissures possessed poor mechanical properties and were not feedable (Table 2); e.g., for the KVA64:PEO (70:30) blend, changing the HME temperature (from 140 °C to 180 °C) generated filaments without fissures and enabled printing. This effect is also visible when comparing their mechanical properties as the filaments’ stress at break (9.1 to 16.8 MPa), strain at break (1.4 to 3.9%) and tensile energy to break (7.2 to 38.0 × 10${}^{4}$ J/m${}^{3}$) augmented in function of the extrusion temperature. A system consisting of Kollidon 12PF and Polyox WSR N10 (70:30) was, however, not feedable, even at higher HME temperatures. Likewise, the mechanical properties of filaments produced at different extrusion temperatures were similar, e.g., the tensile energy to break the filament was 2.4 × 10${}^{4}$ J/m${}^{3}$ for both temperatures. It was noted that the Kollidon 12PF:PEO (70:30) system possessed a large peak in tan δ during a temperature sweep (Figure 8). Such a peak in a blended system indicates energy absorption upon heating, which mostly represents a metastable region in which phase separation occurs [55]. This peak is reduced at a 30:70 ratio between K12PF and PEO, which might explain the inferior stability of the 70:30 K12PF:PEO system.

#### 3.2.3. Moisture Absorption Analysis

The hygroscopicity of the extruded filaments was investigated using Karl–Fischer analysis (Table 3). Immediately after HME, the moisture content of all filaments was rather low (between 0.89 and 1.94%), as was expected due to the absence of solvents during the process and the applied heat within the barrel [16]. After one week of storage in a high moisture environment (RH 60%), the blends had absorbed moisture to different extents. For example, the moisture content of EPO:PEO (70:30) after HME and after storage in a high % RH atmosphere did not differ much (1.39% and 1.56%, respectively). On the other hand, filaments consisting of K12PF:PEO (70:30) and KVA64:PEO (70:30) displayed high hygroscopicity (14.32% and 8.70% moisture content, respectively). It was shown previously that water uptake negatively impacts the mechanical stability of filaments due to the plasticizing effect of water [18,56]. Elasticity modulus and stress at break decreased and strain at break increased for the filaments with a higher moisture content, changing their feeding behaviour. Unfortunately, the printability behaviour also changed, e.g., print temperature had to be changed in function of the storage time at 25 °C/60% RH for a poly(vinyl alcohol)-based filament containing paracetamol (10–50% *w*/*w*) due to a change in the glass transition temperature [56]. Therefore, a low hygroscopicity of the feedstock filament is favorable in terms of stability of the feedability and printability behaviour. Moreover, ZHT was prone to photolytic oxidation (Table 1). As such, a low hygroscopicity of the feedstock would also be beneficial to delay such degradation pathways of the API.

### 3.3. Characterization and Printability of Drug-Loaded Filaments

The EPO:PEO blends seemed most promising to prepare drug-loaded filaments based on the mechanical and rheological properties, combined with the filaments’ low hygroscopicity. The EPO:PEO (70:30) blend was favored over the 30:70 blend due to the faster disintegration time of this formulation, as reported in earlier research [23]. Filaments with either 1% or 10% drug load were prepared using HME.

#### 3.3.1. Mechanical Properties and Content Uniformity

Both 1 and 10% drug-loaded filaments were feedable and printable. However, the addition of ZHT deteriorated the mechanical properties of the filament compared to the placebo EPO:PEO (70:30) blend, as can be seen in Table 2. Stress (14.0, 13.0 and 9.7 MPa) and strain at break (4.3, 2.4 and 2.3%) diminished together with the tensile energy to break the filament (36.0, 13.8 and 11.9 × 10${}^{4}$ J/m${}^{3}$) for the 0, 1 and 10% drug-loaded filaments, respectively. This is consistent with literature findings where, e.g., the addition of metoprolol tartrate (crystalline) in general reduced the mechanical properties [34]. The drug is expected to remain crystalline within the polymer matrix based on the mechanical data, Arrhenius activation energy of flow and processing temperature (160 °C), which is below the melting point of ZHT (188.03 °C).

Both drug-loaded polymer systems exhibited excellent content uniformity: 101.84% ± 2.78% and 98.80% ± 3.85% of the theoretical drug load for 1% or 10% drug loaded filaments, respectively.

#### 3.3.2. Rheological Properties

Qualitative caplets were printed with a nozzle temperature of 160 °C and bed temperature of 55 °C for the placebo and drug-loaded blends. In general, when a crystalline filler or API is added to the blend, the printing temperature needs to be increased [34]. This printing temperature was previously linked to the Arrhenius flow activation energy (Ea) of the matrix system. The Ea is calculated from the shift factors (aT) of individual frequency sweeps when a temperature-invariant mastercurve is constructed [11]. As can be seen in Table 4, the addition of crystalline ZHT to the blend increased the Ea only to a small extent: from 119.51 kJ/mol for the placebo blend to 119.96 kJ/mol and 136.81 kJ/mol for 1% and 10% drug loaded filaments, respectively. This marginal effect most likely results from the low concentration of API present within the drug-loaded filaments and explains why the same printing temperature could be employed for all EPO:PEO (70:30) systems. Furthermore, a drop in initial viscosity (from 1417 to 1010 Pa.s), less shear thinning (0.467 to 0.387) and later onset of shear thinning (3072 to 6309 Pa) was detected when comparing the 10% drug-loaded blends with the placebo blend. As discussed previously [11], it is difficult to correlate these parameters with actual differences in caplet quality or print settings. Moreover, the measurements were conducted on a controlled-stress rheometer, which does not correspond to the actual flow conditions imposed on the polymeric melts in the printer nozzle, where other phenomena (e.g., inertia, turbulent flow conditions, energy dissipation, die swell) might occur.

### 3.4. Caplet Printing and Characterization

A variety of caplets were printed and characterized. Firstly, the influence of nozzle size and % overlap was investigated using caplets with 11 mm length, 4.4 mm height and 5.5 mm width. Secondly, a comparison was made between caplets printed with either the EPO:PEO:ZHT (63:27:10) or EPO:PEO:ZHT (69.3:29.7:1) blend. Thirdly, the effect of caplet size was investigated by comparison of the small caplet with a larger one (19.2 mm × 9.6 mm × 7.68 mm).

#### 3.4.1. Influence of Nozzle Size and Overlap

**Figure 9:**
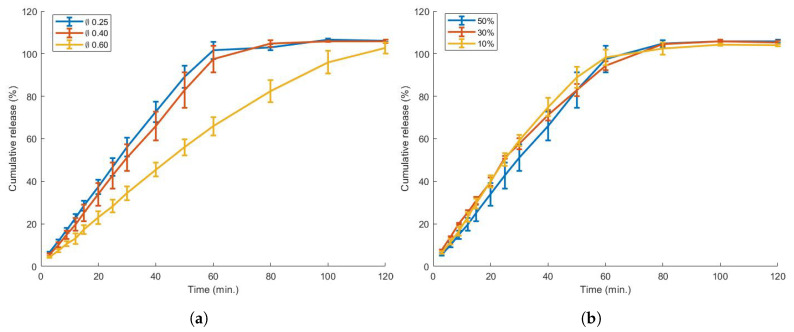
The effect of nozzle size (**a**) and overlap (**b**) on the in vitro dissolution behaviour. A nozzle size of Ø0.60 mm has delayed release compared to Ø0.25mmand 0.40 mm. The % overlap seems to have no significant effect on the release profile.

The effect of printing parameters on the dissolution profile, weight or hardness of 3D-printed tablets has been investigated previously; e.g., increasing the infill of a tablet resulted in a higher dose and longer dissolution time [57,58], while the shell thickness also significantly retarded dissolution rate [59,60]. In the current research, caplets were printed with a nozzle diameter varying between Ø0.25, 0.40 and 0.60 mm. To our knowledge, the effect of this printing parameter on caplet characteristics and visual quality was not yet assessed. A wider nozzle diameter increased the weight of the caplet and, surprisingly, also the weight variability (Table 5); e.g., when caplets were printed with a Ø0.60 mm nozzle instead of a Ø0.25 mm nozzle, the mean weight and relative standard deviation (RSD) increased from 113.16 mg and 0.85 % to 124.20 mg and 3.75 %, respectively. However, even for the Ø0.60 mm caplets, the Ph.Eur specifications were met (max 10% RSD for an average caplet weight <300 mg). Possibly, the FDM printer is able to control the smaller feed rate and polymer stream more accurately with the Ø0.25 mm nozzle. Higher hardness values were also measured when increasing the nozzle diameter: a diametrical crushing force of 17.5, 23.1 and 29.3 N was detected for caplets manufactured using nozzles with a diameter of 0.25, 0.4 and 0.6 mm, respectively. This effect is linked to a larger nozzle diameter resulting in an increased road width, thus generating less interfaces between polymer beads, which are the most fragile parts of the 3D-printed caplet [11]. The effect of the nozzle size on the in vitro dissolution profile can be seen in Figure 9. While a caplet produced with a Ø0.6 mm nozzle showed a delayed dissolution profile, both caplets from Ø0.25 mm and 0.4 mm nozzles showed a similar profile. This similarity in dissolution profile could be linked to the porosity (Table 5): Ø0.25 mm and 0.4 mm caplets possess similar porosities (59.7 and 60.9%, respectively), in contrast to a porosity of 48.6% for caplets manufactured using a Ø0.6 mm nozzle. It was shown previously that a lower porosity prolonged the disintegration time [61]. As such, porosity might provide a simple, fast screening tool for the dissolution behaviour of 3D-printed tablets. The % overlap did not seem to considerably alter the release profile.

**Figure 10:**
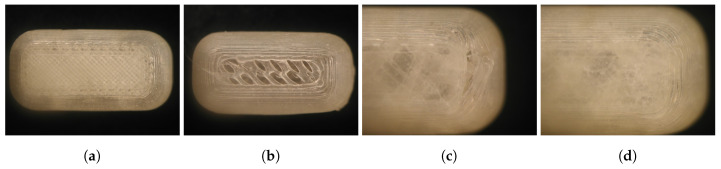
The effect of nozzle size and overlap on the visual quality of the resulting caplet. (**a**) A detailed caplet printed using a nozzle of Ø0.25 mm; (**b**) a caplet printed using a nozzle of Ø0.60 mm showing a less detailed surface; (**c**) a caplet produced with an overlap of 10% with visual deformation between infill and shells; and (**d**) a caplet printed with an overlap of 50%.

The effect of these properties (nozzle size, % overlap) on the visual quality of the resulting caplet is displayed in Figure 10. A caplet printed with a Ø0.25 mm nozzle size and 50% overlap was chosen as the preferred system. A smaller nozzle diameter provides a more detailed caplet, and an overlap of 50% ensures the absence of printing defects, while these settings do not delay the dissolution time of the caplet.

#### 3.4.2. Influence of Drug Load and Caplet Size

The ultimate goal is to prepare a flexible dosing platform providing a drug dose of maximally 10 mg, after which a gradual decrease in dose is initialized. A 1% drug-loaded filament could be used to provide such flexibility: an 11 × 5.5 × 4.4 caplet was seen to be the smallest size feasible to print, while caplets with 19.2 mm × 9.6 mm × 7.68 mm dimensions and 90% infill were seen as the largest acceptable size. Both caplet sizes were printed and analysed, as can be seen in Table 6. It was demonstrated that caplets containing <1 mg and up to 10 mg zolpidem could be printed with the same filament, achieving an acceptable standard deviation of the caplets size and weight (2.24 and 2.50%, respectively).

**Figure 11:**
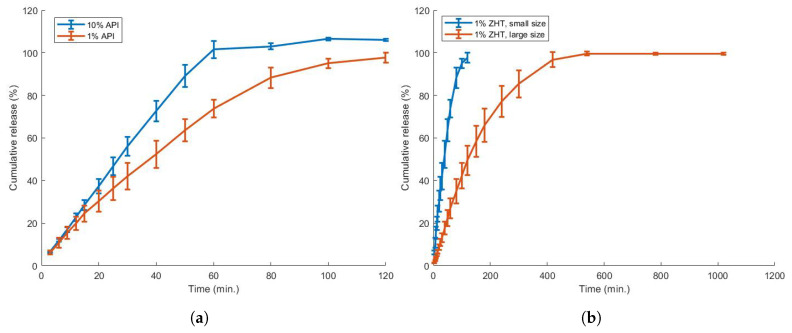
The effect of ZHT drug load (**a**) and caplet size (**b**) on the in vitro dissolution behaviour. Decreased drug load resulted in a decreased in vitro dissolution rate, while enlargement of the caplet greatly affected the dissolution profile.

The dissolution profile of both caplets is presented in Figure 11. The smallest caplet achieved >80% drug release within ±60 min, while it took more than 200 min to dissolve >80% of the largest caplet. The effect of drug load was also investigated by comparing the smallest sized caplets of 1 and 10% drug loaded filament: although a faster dissolution rate (>80% within 45 min.) was achieved using the 10% zolpidem filament, the USP limit of >80% release within 15 min was not achieved.

### 3.5. Enhancing the Dissolution Rate

An attempt was made to increase the dissolution rate of the EPO:PEO:ZHT (69.3:29.7:1) caplets by either modifying the caplet geometry or by the incorporation of a disintegrant.

#### 3.5.1. Caplet Modification

**Figure 12:**
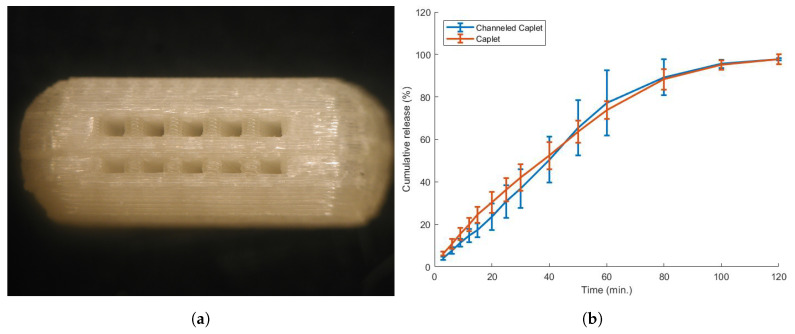
(**a**) The effect of caplet modification on the dissolution behaviour of the EPO:PEO:ZHT blend (69.3:29.7:1). Two rows with five columns of channels (0.6 mm size) were added to the caplet. (**b**) These modifications did not significantly alter the dissolution profile of the caplet.

A caplet containing two rows of five channels was designed as can be seen in Figure 12. This modification marginally increased weight variation but did not alter the dimensional deviations in comparison with non-channeled tablets (Table 7). Unfortunately, this adaptation did not improve the dissolution characteristics of the investigated caplet (Figure 12). In the literature, however, such an adaptation proved to be successful for hydrochlorothiazide caplets [62]. In their work, channels of 0.6 mm size on the short side of the caplet accelerated drug release substantially for caplets with 100% infill. Most likely, the channels are not effective for the zolpidem caplets due to their low infill value (20%). The printer needs to completely fill the layers above and below the channels in order to achieve a qualitative caplet. As a result, the channeled caplets have an overall higher infill value compared to non-channeled caplets, and it was shown already that an increased infill reduces dissolution speed [57,58,63]. This increased overall infill was confirmed by the weight of the channeled caplets, which was higher than the non-channeled caplets, e.g., 132.8 mg compared to 93.9 mg. In conclusion, incorporation of channels was not effective for low infill caplets.

#### 3.5.2. Formulation Modification

**Figure 13:**
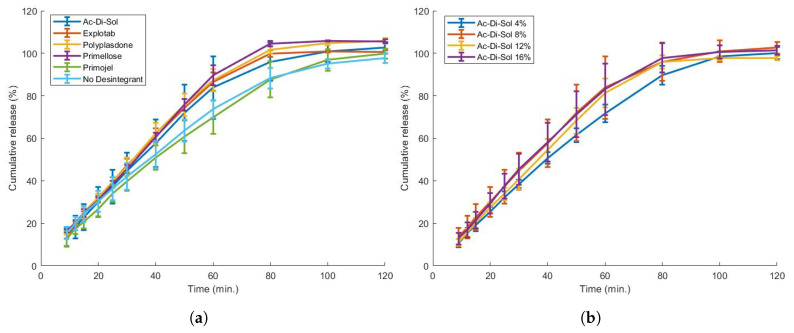
(**a**) The effect of disintegrant addition on the dissolution behaviour of the EPO:PEO:ZHT blend (69.3:29.7:1). Five different disintegrants were added in an 8% ratio. The addition of disintegrant seemed to marginally increase the dissolution speed, except for Primojel. (**b**) An increase in disintegrant concentration did not significantly alter the dissolution behaviour.

The addition of a disintegrant to the formulation often accelerates drug release. Five disintegrants (Ac-Di-Sol, Explotab, Polyplasdone, Primojel and Primellose) were added to the formulation in a high percentage (8%). Such formulations could be printed successfully (Table 7), but yielded only a minor, almost insignificant increase of drug dissolution speed, except for Primojel which did not alter the dissolution profile (Figure 13). This behaviour for 3D-printed formulations containing disintegrants was described previously [62,64]. In literature, the addition of disintegrants up to 10% did not alter the dissolution speed significantly [64] or even interfered with filament feedability [62]. This can be explained by the fact that disintegrants mostly work by increasing the tablet pressure by swelling or wicking, which is hampered in formulations containing a high polymer content [64].

The concentration of one disintegrant (Ac-Di-Sol) was varied to assess the concentration effect. A higher disintegrant content improved feedability behaviour (Table 2). A filament containing 16% Ac-Di-Sol required more energy (175.1 × $10^{4}$ J/m${}^{3}$) to break the filament compared to 4% Ac-Di-Sol (15.8 × $10^{4}$ J/m${}^{3}$) and even showed a ductile instead of a brittle behaviour. This contradicts earlier research, where impaired feedability at a higher disintegrant concentration was reported [62]. While quantitative caplets could be printed (Table 7), this did not significantly improve the dissolution rate of the caplets (Figure 13).

Future steps will include the addition of soluble fillers which have shown to act as pore-formers (e.g., mannitol) in 3D-printable formulations to enable immediate release of the proposed caplets [65,66].

## 4. Conclusions

This study highlighted the feasibility of extrusion-based 3D printing in the production of personalised dosage forms for zolpidem withdrawal therapy. It was discovered that ZHT undergoes a polymorphic transition from form A to C upon heating and was especially prone to photolytic oxidation, highlighting the need for appropriate packaging of the 3D-printed caplets. The addition of Polyox WSR N10 to hydrophilic pharmaceutical polymers in a 30:70 or 70:30 ratio enhanced their mechanical properties and enabled their printing, although large viscosity differences limited the processability range. An EPO:PEO:ZHT (69.3:29.7:1) blend was an excellent matrix, with suitable mechanical/rheological properties for 3D printing and low hygroscopicity favoring feedstock and ZHT stability. A flexible dosing platform capable of printing caplets with a dose ranging from <1 to 10 mg was constructed. Such flexible doses could be used to develop a highly personalised withdrawal scheme based on the patient’s specific situation (e.g., duration of use, start dose…) and could easily be adapted during withdrawal therapy. This study also highlighted that nozzle diameter and % overlap are important parameters in terms of caplet quality, while they marginally influence the dissolution profile. Tablet porosity might be employed to predict dissolution behaviour. The addition of disintegrants and caplet modification were investigated as tools to enhance dissolution speed, but were not deemed effective for low-infill extrusion-based 3D printed caplets.

## Figures and Tables

**Table 1:** HPLC-UV analysis of zolpidem hemitartrate samples subjected to photolytic degradation. In its solid state, zolpidem was stored in either a hermetically sealed glass vial or at 40% RH. The % recovery can be found between brackets.

	Solution				Solid State	
**Peak**	**Light**	**Dark**	**Light**	**Dark**	**40% RH**	**Sealed**
**RRT**	**7 days**	**7 days**	**3 days**	**3 days**	**7 days**	**3 months**
0.69	7.2%	/	0.1%	/	0.6%	0.2%
0.75	4.0%	/	/	/	/	/
0.85	5.5%	/	0.1%	/	/	/
1	78.7%	100%	99.7%	100%	99.4%	99.8%
	(58.1%)	(96.6%)	(94.2%)	(99.8%)	(96.0%)	(96.9%)
2.05	4.6%	/	0.2%	/	/	/

**Table 2:** Mechanical properties of all filaments analysed: Young’s modulus (EY), stress and strain at breaking point and tensile energy to break. Non-printable filaments are indicated with an asterix (*).

Blend	Young’s Modulus MPa	Stress at Break MPa	Strain at Break %	Energy to Break ×$10^{4}$ J/m${}^{3}$
	**Placebo filaments**			
K12PF:PEO (70:30)(120) *	722.0 ± 63.1	3.5 ± 0.7	0.7 ± 0.2	2.4 ± 0.8
K12PF:PEO (70:30)(160) *	401.2 ± 26.2	3.8 ± 0.9	0.8 ± 0.2	2.4 ± 0.6
K12PF:PEO (30:70)(140)	507.2 ± 105.2	14.0 ± 1.4	3.3 ± 0.3	26.0 ± 4.8
K12PF:PEO (30:70)(160)	448.9 ± 140.7	14.1 ± 1.6	3.6 ± 0.5	28.0 ± 7.0
KVA64:PEO (70:30)(140) *	859.7 ± 60.4	9.1 ± 1.4	1.4± 0.2	7.2 ± 2.0
KVA64:PEO (70:30)(180)	714.1 ± 92.1	16.8 ± 2.1	3.9 ± 0.3	38.0 ± 5.7
KVA64:PEO (30:70)(160)	531.5 ± 22.1	17.8 ± 0.5	4.3 ± 0.5	42.0 ± 6.5
SOL:PEO (70:30)	649.0 ± 34.8	11.7 ± 3.3	2.3 ± 0.8	15.7 ± 5.8
SOL:PEO (30:70)	400.8 ± 58.3	16.5 ± 3.2	5.8 ± 0.6	57.8 ± 9.1
EPO:PEO (70:30)	400.1 ± 33.4	14.0 ± 0.6	4.3 ± 0.5	36.0 ± 5.3
EPO:PEO (30:70)	754.1 ± 132.3	20.9 ± 0.9	4.9 ± 0.3	57.0 ± 10.1
	**Drug-loaded filaments**			
EPO:PEO:ZHT (69.3:29.7:1)	522.1 ± 120.1	13.0 ± 1.7	2.4 ± 0.5	13.8 ± 3.4
EPO:PEO:ZHT (63:27:10)	517.2 ± 47.0	9.7 ± 1.1	2.3 ± 0.3	11.9 ± 2.1
	**Drug-loaded filaments with disintegrant**			
	**EPO:PEO:ZHT 69.3:29.7:1**			
AcDiSol (4%)	473.0 ± 159.6	11.2 ± 2.6	2.6 ± 0.7	15.8 ± 9.3
AcDiSol (8%)	744.0 ± 129.6	10.3 ± 2.7	1.6 ± 0.6	9.6 ± 6.3
AcDiSol (12%)	577.1 ± 151.2	13.2 ± 2.0	2.9 ± 0.8	21.7 ± 8.7
AcDiSol (16%)	565.4 ± 132.2	12.3 ± 0.7	13.8 ± 2.6	175.1 ± 32.8
Explotab (8%)	442.20 ± 125.2	12.1 ± 3.4	3.8 ± 1.6	28.7 ± 7.4
Polyplasdone (8%)	539.3 ± 145.4	10.0 ± 2.1	2.0 ± 0.7	10.9 ± 5.9
Primojel (8%)	795.3 ± 125.5	11.2 ± 3.2	1.7 ± 0.6	11.2 ± 7.0
Primellose (8%)	245.5 ± 57.1	12.7 ± 1.8	5.8 ± 0.4	43.5 ± 6.0

**Table 3:** Moisture content of filaments directly after extrusion and after storage in a 60% RH environment for 7 days.

Blend	Moisture Content	
	After Extrusion	7d at RH 60%
	%	%
K12PF:PEO (70:30)	1.94	14.32
K12PF:PEO (30:70)	1.20	6.83
KVA64:PEO (70:30)	1.54	8.70
KVA64:PEO (30:70)	1.20	4.28
SOL:PEO (70:30)	1.27	4.63
SOL:PEO (30:70)	1.24	3.08
EPO:PEO (70:30)	1.39	1.56
EPO:PEO (30:70)	0.89	1.63

**Table 4:** Cross-model parameters and Arrhenius activation energy of flow (kJ/mol) for the placebo and drug-loaded filaments.

Blend	Cross-Model Parameters			
	$\eta_{0}$ (Pa × s)	$\tau^{*}$ (Pa)	n	Ea (kJ/mol)
EPO:PEO (70:30)	1417	3072	0.467	119.51
EPO:PEO:ZHT (69.3:29.7:1)	1432	5641	0.426	119.96
EPO:PEO:ZHT (63:27:10)	1010	6309	0.387	136.81

**Table 5:** The effect of the nozzle size used during 3D printing on a variety of caplet properties.

Nozzle Size	Weight	Length	Width	Height	Hardness	Porosity
Ø	mg	mm	mm	mm	N	%
0.25	113.16 ± 0.96	10.90 ± 0.02	5.39 ± 0.02	4.44 ± 0.03	17.50 ± 2.80	59.65 ± 1.51
0.40	118.34 ± 2.36	10.76 ± 0.06	5.35 ± 0.03	4.49 ± 0.02	23.10 ± 1.91	60.86 ± 0.68
0.60	124.20 ± 4.66	10.75 ± 0.03	5.42 ± 0.02	4.57 ± 0.09	29.30 ± 2.91	48.56 ± 2.75

**Table 6:** The effect of caplet size (as constructed in the .stl file) on the final caplet characteristics for the EPO:PEO:ZHT (69.3:29.7:1) blend printed with a Ø0.25 mm nozzle.

Size	Weight	Length	Width	Height
mm	mg	mm	mm	mm
11.0 × 5.5 × 4.4	93.90 ± 2.10	10.87 ± 0.08	5.36 ± 0.01	4.36 ± 0.02
19.2 × 9.6 × 7.68	1028.10 ± 25.70	19.04 ± 0.06	9.44 ± 0.02	7.55 ± 0.12

**Table 7:** Weight and dimension analysis of modified caplets in order to achieve immediate release dosage forms. Caplets (11.0 × 5.5 × 4.4 mm) are modified by either channel incorporation in an EPO:PEO:ZHT (69.3:29.7:1) blend or by formulation modification (inclusion of a disintegrant).

Blend	Weight mg	Length mm	Width mm	Height mm
Channeled caplets	132.8 ± 5.0	10.80 ± 0.01	5.40 ± 0.02	4.24 ± 0.01
AcDiSol (4%)	103.6 ± 2.8	10.88 ± 0.02	5.39 ± 0.01	4.32 ± 0.02
AcDiSol (8%)	104.0 ± 3.7	10.89 ± 0.03	5.42 ± 0.02	4.32 ± 0.03
AcDiSol (12%)	107.2 ± 3.6	10.89 ± 0.02	5.41 ± 0.02	4.32 ± 0.04
AcDiSol (16%)	108.2 ± 1.8	10.86 ± 0.04	5.41 ± 0.01	4.33 ± 0.03
Explotab (8%)	115.0 ± 3.1	10.91 ± 0.04	5.43 ± 0.01	4.36 ± 0.03
Polyplasdone (8%)	99.6 ± 3.1	10.88 ± 0.03	5.42 ± 0.02	4.32 ± 0.03
Primojel (8%)	113.7 ± 4.7	10.91 ± 0.03	5.42 ± 0.02	4.36 ± 0.04
Primellose (8%)	91.0 ± 3.2	10.81 ± 0.03	5.35 ± 0.01	4.27 ± 0.02

## Data Availability

Not applicable.

## Supplementary Materials

The following are available online at https://www.mdpi.com/article/10.3390/pharmaceutics13101684/s1, Video S1: Melting of lidocaine hydrochloride monohydrate visualised using hot stage microscopy, Video S2: Heating of zolpidem hemitartrate visualised using hot stage microscopy.
